# Supplementary material for: Mitochondrial genetic diversity, selection and recombination in a canine transmissible cancer
Source: eLife. 2016 May 17;5:e14552. doi: 10.7554/eLife.14552 (PMC4869914; doi:10.7554/eLife.14552)
Supplement: Figure 1—source data 1. — Maximum likelihood phylogenetic tree constructed using 449 complete CTVT mitochondrial genomes and 590 complete dog mitochondrial genomes. All sequences are labelled with sample identifier, country, breed and haplotype name. The sample identifier for CTVT hosts is the sample name (Supplementary file 1), the sample identifier for the publicly available dogs is the accession number. Scale bar indicates base substitutions per site. DOI: http://dx.doi.org/10.7554/eLife.14552.004 [file elife-14552-fig1-data1.pdf]

Figure 1 - source data 1

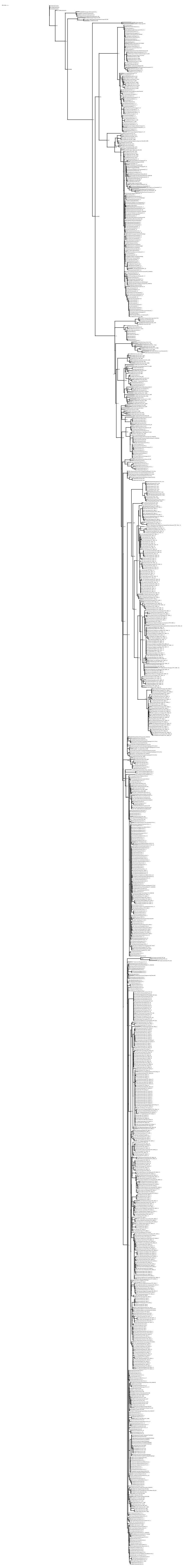

Figure 1 - source data 1. Maximum likelihood phylogenetic tree of CTVT mtDNA. Maximum likelihood phylogenetic tree constructed using 449 complete CTVT mitochondrial genomes and 590 complete dog mitochondrial genomes. All sequences are labelled with sample identifier, country, breed and haplotype name. The sample identifier for CTVT hosts is the sample name (Supplementary file 1), the sample identifier for the publicly available dogs is the accession number. Scale bar indicates base substitutions per site.
