# Supplementary material for: Mitochondrial genetic diversity, selection and recombination in a canine transmissible cancer
Source: eLife. 2016 May 17;5:e14552. doi: 10.7554/eLife.14552 (PMC4869914; doi:10.7554/eLife.14552)
Supplement: Figure 1—source data 2. — Maximum likelihood phylogenetic trees for CTVT mtDNA in (A) clade 1 (n = 170) (B) clade 2 (n = 252) (C) clade 3 (n = 22) (D) clade 4 (n = 3) and (E) clade 5 (n = 2), rooted with haplotypes CTVT1 to CTVT5 respectively, which contain clade-defining germline and potential somatic substitutions specific to each clade (Figure 1—figure supplement 4). Bootstrap values were calculated from 100 bootstrap replicates and are shown where bootstrap values ≥60. Scale bars indicate base substitutions per site. Clade 5 contains only two tumours, which are identical both to each other and to the CTVT5 haplotype; thus the tree for this clade was created separately and does not have a scale bar. DOI: http://dx.doi.org/10.7554/eLife.14552.005 [file elife-14552-fig1-data2.pdf]

Figure 1 - source data 2

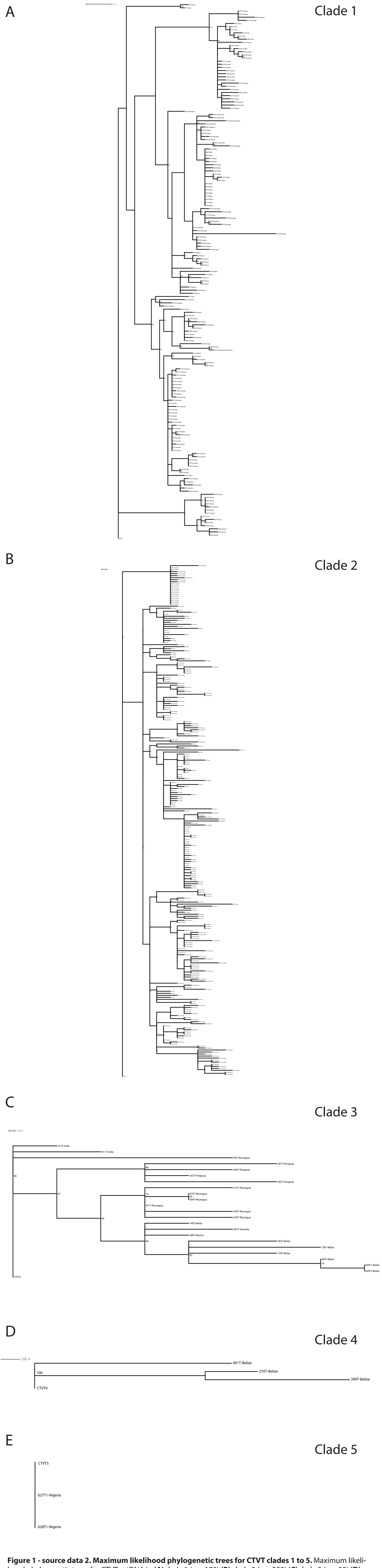

Figure 1 - source data 2. Maximum likelihood phylogenetic trees for CTVT clades 1 to 5. Maximum likelihood phylogenetic trees for CTVT mtDNA in **(A)** clade 1 (n = 170) **(B)** clade 2 (n = 252) **(C)** clade 3 (n = 22) **(D)** clade 4 (n = 3) and **(E)** clade 5 (n = 2), rooted with haplotypes CTVT1 to CTVT5 respectively, which contain clade-defining germline and potential somatic substitutions specific to each clade (Figure 1-figure supplement 4). Bootstrap values were calculated from 100 bootstrap replicates and are shown where bootstrap values ≥ 60. Scale bars indicate base substitutions per site. Clade 5 contains only two tumours, which are identical both to each other and to the CTVT5 haplotype; thus the tree for this clade was created separately and does not have a scale bar.
