# Supplementary material for: Mitochondrial genetic diversity, selection and recombination in a canine transmissible cancer
Source: eLife. 2016 May 17;5:e14552. doi: 10.7554/eLife.14552 (PMC4869914; doi:10.7554/eLife.14552)
Supplement: Figure 3—source data 1. — Maximum likelihood cladograms constructed using clade 1 mtDNA positions (A) 1-5429bp and (B) 5430-16176bp (see Materials and methods). Trees were constructed with 153 clade 1 CTVT mtDNAs rooted with the CTVT1 haplotype, which contains clade 1 clade-defining germline and potential somatic substitutions (Materials and methods, Figure 1—figure supplement 4). Bootstrap values were calculated from 100 bootstrap replicates and are shown where bootstrap values ≥60. DOI: http://dx.doi.org/10.7554/eLife.14552.014 [file elife-14552-fig3-data1.zip › 14552_Figure_3-source_data_1.pdf]

Figure 3 - source data 1

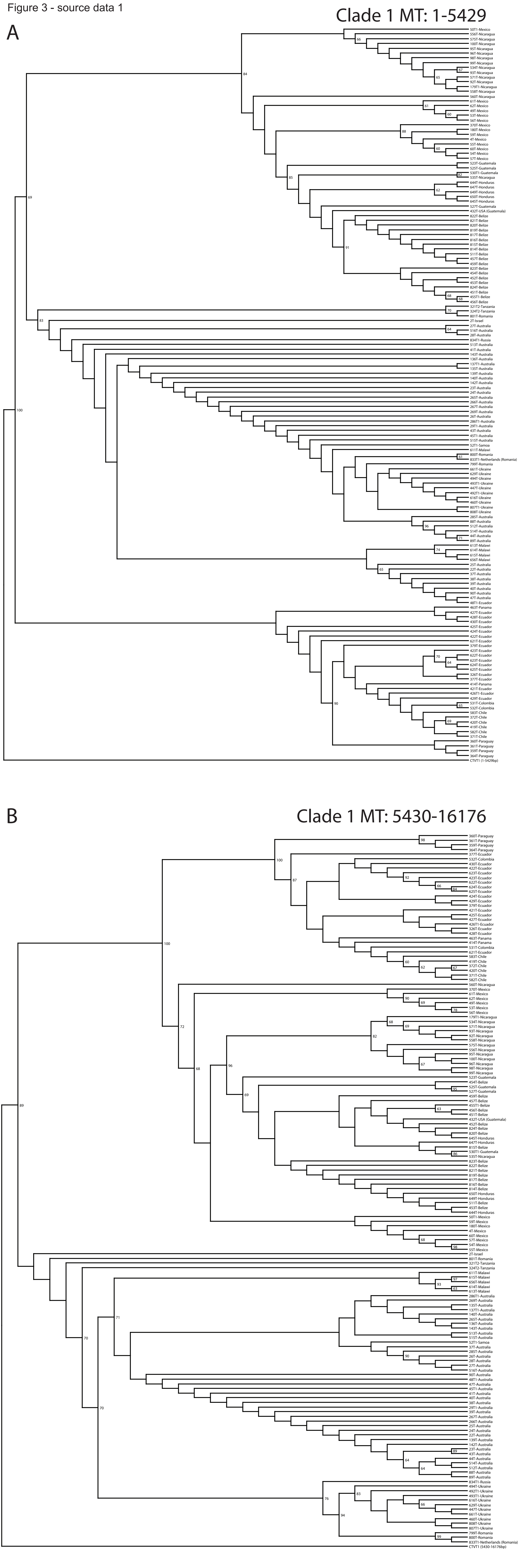

Figure 3 - source data 1. Ancient mtDNA recombination in CTVT clade 1. Maximum likelihood cladograms constructed using clade 1 mtDNA positions **(A)** 1-5429bp and **(B)** 5430-16176bp (see Methods). Trees were constructed with 153 clade 1 CTVT mtDNAs rooted with the CTVT1 haplotype, which contains clade 1 clade-defining germline and potential somatic substitutions (Methods, Figure 1-figure supplement 6). Bootstrap values were calculated from 100 bootstrap replicates and are shown where bootstrap values ≥ 60.
